# A Consultation at the Polyclinic

**Published:** 1900-05-12

**Authors:** J. Seymour Taylor


### Hospital Clinics and Medical Progress.

### A CONSULTATION AT THE POLYCLINIC.

By J. Seymour Taylor, M.D., F.R.C.P.

Specially reported for The Hospital, and corrected by the Author.

**A Case of Disease of the Pons Varolii with Right Hemiplegia.**—This case, as you will see from the gait, is one of right-sided hemiplegia. The patient is a man 33 years of age, and by occupation a road-sweeper, and gives no history for our guidance. There is no history of syphilis nor of tuberculous disease in the family, nor are there any signs of tuberculosis about his lungs or elsewhere. Five years ago he complained that he had double vision, especially in the left eye, but he did not test which eye he saw double with, but if you shut the right eye up he sees with the left eye two images. So that he has diplopia on the left side; that form of diplopia in which a double vision is on the same side as there is palsy, *e.g.*, homonymous. In August, 1899, *i.e.*, eight months ago, his right leg began to be weak, and he began to suffer from loss of power in the right arm.

**Present Condition.**—Paresis of the right side, arm, and leg. The foot scrapes the ground as he walks, and in consequence he wears out the toe-piece of his boot before any other part. You may see this characteristic gait of the hemiplegic commonly enough in the London streets. There is also on the left, affection of the sixth nerve, the seventh slightly, and the eighth, *i.e.*, he is deaf in the left ear. Therefore the case comes under the category of crossed paralysis, *i.e.*, paralysis of one cranial nerve (or more) on one side, with the loss of power in arm and leg on the opposite side. The reason that a patient suffers from this form of paralysis is due to the fact that the fibres going to the face and eye cross at a higher level in the cerebro-spinal axis than those fibres that supply the arm and leg. His grasp is feebler in the right than in the left arm; but from the fact that there is no defect in speech, you gather that his lesion is not in Broca's area, but in the lower part of the cerebro-spinal tract—the pons and medulla. Then comes the question, What are his hemiplegic symptoms? They are the loss of power already mentioned, the wasting, and the increased deep reflexes. The latter are so increased that he is brought to the ground if he catches his toe against anything as he walks along. Ankle-clonus and patellar-clonus are present, but not so easy to elicit. These symptoms must be taken as showing descending lesion of the crossed pyramidal tract, and secondary to some lesion at a higher level in the cerebro-spinal axis. Crossed paralysis may occur either from a lesion in the crus, the pons, or in the medulla. Here we may infer that the crus remains intact because there is no affection of the third nerve: for the third nerve, as you know, arises superficially from between the two crura close under the middle line, and consequently any lesion of the crus would be bound to affect both the third nerves. If he has medullary disease it is probably secondary to pressure of whatever is causing the trouble, thus affecting the twelfth nerve. The disease is not in the nuclei in the floor of the fourth ventricle for the simple reason that the pair of the sixth nerves would be affected, since their nuclei are so close to the middle line that it is impossible to conceive an injury not affecting both nerves. One would also expect the third nerves on both sides to be similarly affected. The concomitant muscle of the external rectus on the left side is the internal rectus of the right, and, if the nucleus of the sixth were affected, you would have the third nerve on the opposite side paralysed, and as a result the right eye would be deviated from the middle line. You will remember this better by thinking of the manipulation of the reins in driving a pair of horses, where the guidance is similar to that of the eyes. Thirdly, does the lesion occur in the pons? Yes, it does. Pontine disease will account for the symptoms, and the part affected is the lower portion of the pons, because the sixth, seventh, and eighth nerves are affected, and only those; if the upper portion were diseased, the fifth nerve would not be intact, as it is in this man.

Does the lesion—whatever it may be—go deep into the pons and into the tegmental fibres? No, it does not; for if it did there would be loss of sensation in the arms, face, and legs; this symptom is absent in this man. It is quite possible that the patient has slight affection of the twelfth nerve; for at one time the tongue was thought to be protruded to the left side.

Having discussed the site of the lesion, the question is now as to the nature of the growth? There is no history of injury to point to the possibility of the existence of hæmorrhage. There is the possibility of a new growth. I cannot say what its nature is; it may be glioma, it may be a caseous tubercular mass. In the possible event of its being specific, we treated the man with iodide of potassium and biniodide of mercury in fairly large doses. Under these drugs he has slightly improved. In these doubtful and unknown cases we always give iodide of potassium. I intend to continue giving mercury in constant small doses with large doses of iodide of potassium (10-20 grains per diem). If I were sure of the lesion having a specific origin I should order half salt-spoonful doses of iodide of potassium at a time; it might be poured into his drink at meals. He should not have less than a drachm of iodide of potassium a day.

The difficulty in this case is not one of anatomical diagnosis so much as the actual cause of the trouble.

**Case of Jaundice.**—This man was sent up to my wards from the country as a case for diagnosis, and I thought I could not do better than bring him here this afternoon. He has for some years lived in the tropics, and has been a free drinker; but, according to his own account, he has not indulged to excess. For some months he has complained of loss of appetite and flesh; afterwards he had some difficulty in swallowing, and jaundice supervened. As a proof of his emaciation you have only to observe that the xiphoid cartilage preserves the shape of the arc that a distended abdomen always possesses. This curved ensiform cartilage is a great guide as to the amount of emaciation which has taken place in the belly. It was observed originally by a physician (Dr. Ord) in the wards of St. Thomas's, and I have myself verified its accuracy from time to time. The jaundice is due to obstruction, *i.e.*, bile does not enter the duodenum or the gastro-enteric canal because the motions are pale and offensive. He has dysphagia specially marked when he tries to eat solids, so there is some obstruction in his œsophagus. I would impress upon you one point of importance in connection with jaundice; everybody emaciates after jaundice, therefore always ask the patient if he emaciated before the jaundice came on. In the case under notice the emaciation preceded the jaundice; when this is so you have strong presumptive evidence of a new growth, causing the emaciation with jaundice as a secondary complication. On passing the œsophageal bougie 15 inches from the incisor teeth, I came across an obstruction, *i.e.*, an obstruction at the point where the œsophagus enters the cardiac end of the stomach. The patient was under my care for three weeks, and is now an out-patient. With these symptoms there can be only one diagnosis, viz., existence of a neoplasm involving the cardiac end of the stomach, and the secondary infiltration of glands, causing obstruction to the hepatic duct, or the cystic or common bile duct. Although the patient thinks the jaundice is not quite so marked as it was, my experience is that jaundice due to malignant disease of the liver never remits; and he is quite as yellow now as he ever was.

**Treatment.**—There is a prescription which I strongly advocate; how it acts I do not know. You may call this empiricism, but then I think much of our treatment is empirical. The prescription to which I refer is many years old, and is one of Addison's, I believe. The iodide of potassium was added by Brinton.

| | |
|---|---|
| ℞ Sodæ bicarb. ... ... ... | gr. xv. |
| Acid. hydrocyanic dil. ... ... | ♏ iii. |
| Sp. ammon. arom. ... ... ... | ♏ x. |
| Potassii iodidum ... ... ... | gr. iii. |
| Inf. gent. co. ad. ... ... ... | ℥ i. |

This mixture relieves the patient wonderfully, and diminishes the catarrh of the stomach by increasing the secretion of hydrochloric acid. It is taken three times a day.

